# Evaluation of Social Determinants of Health and Prostate Cancer Outcomes Among Black and White Patients: A Systematic Review and Meta-analysis

**DOI:** 10.1001/jamanetworkopen.2022.50416

**Published:** 2023-01-11

**Authors:** Randy A. Vince, Ralph Jiang, Merrick Bank, Jake Quarles, Milan Patel, Yilun Sun, Holly Hartman, Nicholas G. Zaorsky, Angela Jia, Jonathan Shoag, Robert T. Dess, Brandon A. Mahal, Kristian Stensland, Nicholas W. Eyrich, Mariana Seymore, Rebecca Takele, Todd M. Morgan, Matthew Schipper, Daniel E. Spratt

**Affiliations:** 1 Department of Urology, University Hospitals Seidman Cancer Center, Case Western Reserve University, Cleveland, Ohio; 2 Department of Biostatics, University of Michigan, Ann Arbor; 3 University of Michigan, Ann Arbor; 4 Central Michigan University School of Medicine, Mt Pleasant; 5 University of Michigan School of Medicine, Ann Arbor; 6 Department of Population Quantitative Health Sciences, Case Western Reserve University, Cleveland, Ohio; 7 Department of Radiation Oncology, University Hospitals Seidman Cancer Center, Case Western Reserve University, Cleveland, Ohio; 8 Department of Radiation Oncology, University of Michigan, Ann Arbor; 9 Department of Radiation Oncology, University of Miami, Miami, Florida; 10 Department of Urology, University of Michigan, Ann Arbor; 11 Department of Urology, Emory University School of Medicine, Atlanta, Georgia; 12 Department of General Surgery, Albany Medical College, Albany, New York

## Abstract

**Importance:**

As the field of medicine strives for equity in care, research showing the association of social determinants of health (SDOH) with poorer health care outcomes is needed to better inform quality improvement strategies.

**Objective:**

To evaluate the association of SDOH with prostate cancer–specific mortality (PCSM) and overall survival (OS) among Black and White patients with prostate cancer.

**Data Sources:**

A MEDLINE search was performed of prostate cancer comparative effectiveness research from January 1, 1960, to June 5, 2020.

**Study Selection:**

Two authors independently selected studies conducted among patients within the United States and performed comparative outcome analysis between Black and White patients. Studies were required to report time-to-event outcomes. A total of 251 studies were identified for review.

**Data Extraction and Synthesis:**

Three authors independently screened and extracted data. End point meta-analyses were performed using both fixed-effects and random-effects models. The Preferred Reporting Items for Systematic Reviews and Meta-analyses (PRISMA) reporting guideline was followed, and 2 authors independently reviewed all steps. All conflicts were resolved by consensus.

**Main Outcomes and Measures:**

The primary outcome was PCSM, and the secondary outcome was OS. With the US Department of Health and Human Services Healthy People 2030 initiative, an SDOH scoring system was incorporated to evaluate the association of SDOH with the predefined end points. The covariables included in the scoring system were age, comorbidities, insurance status, income status, extent of disease, geography, standardized treatment, and equitable and harmonized insurance benefits. The scoring system was discretized into 3 categories: high (≥10 points), intermediate (5-9 points), and low (<5 points).

**Results:**

The 47 studies identified comprised 1 019 908 patients (176 028 Black men and 843 880 White men; median age, 66.4 years [IQR, 64.8-69.0 years]). The median follow-up was 66.0 months (IQR, 41.5-91.4 months). Pooled estimates found no statistically significant difference in PCSM for Black patients compared with White patients (hazard ratio [HR], 1.08 [95% CI, 0.99-1.19]; *P* = .08); results were similar for OS (HR, 1.01 [95% CI, 0.95-1.07]; *P* = .68). There was a significant race-SDOH interaction for both PCSM (regression coefficient, −0.041 [95% CI, –0.059 to 0.023]; *P* < .001) and OS (meta-regression coefficient, −0.017 [95% CI, –0.033 to –0.002]; *P* = .03). In studies with minimal accounting for SDOH (<5-point score), Black patients had significantly higher PCSM compared with White patients (HR, 1.29; 95% CI, 1.17-1.41; *P* < .001). In studies with greater accounting for SDOH variables (≥10-point score), PCSM was significantly lower among Black patients compared with White patients (HR, 0.86; 95% CI, 0.77-0.96; *P* = .02).

**Conclusions and Relevance:**

The findings of this meta-analysis suggest that there is a significant interaction between race and SDOH with respect to PCSM and OS among men with prostate cancer. Incorporating SDOH variables into data collection and analyses are vital to developing strategies for achieving equity.

## Key Points

**Question:**

Is the interaction between race and social determinants of health (SDOH) associated with racial disparities in prostate cancer outcomes?

**Findings:**

This meta-analysis of 47 studies and 1 019 908 patients compared prostate cancer–specific mortality (PCSM) and overall survival between Black and White men. In studies with minimal accounting for SDOH, Black patients had significantly higher PCSM than White patients; however, Black men had significantly lower PCSM in studies with greater accounting for SDOH.

**Meaning:**

This study suggests that the interaction between race and SDOH is significantly associated with racial disparities in health care; collecting and assessing SDOH variables in research may help to create strategies that increase health equity.

## Teaser

This systematic review and meta-analysis evaluates the association of social determinants of health with prostate cancer–specific mortality and overall survival among Black and White patients with prostate cancer.

## Introduction

Racism affects society, health care, and the health outcomes of patients. The societal inequities that impact health outcomes are termed *social determinants of health* (SDOH), which the Centers for Disease Control and Prevention define as “conditions in the places where people live, learn, work and play that affect a wide range of health and quality-of-life-risk and outcomes.”^1^ Common examples of SDOH include income, access to health care, insurance, education, diet, literacy, job opportunity, transportation, neighborhood, and environment. Disparities in SDOH have downstream consequences on health outcomes and are associated with differences in survival between Black and White men in the US.^2,3,4^

Prostate cancer is not only the most commonly diagnosed cancer among men, but it also carries with it one of the largest racial disparities in outcomes in oncology.^5^ There has been a substantial research investment to identify biological causes for these racial disparities, yet data to support specific biological factors associated with these racial differences remain limited.^6,7^ In contrast, Black men with prostate cancer in the US consistently have disparities in SDOH, such as decreased access to health care, reduced prostate-specific antigen screening, economic instability, receipt of less guideline-concordant cancer care, greater number of comorbid conditions, lower likelihood of treatment of comorbid conditions, reduced curative-intent treatment, and reduced access to high-volume centers, all of which reflect SDOH that can be associated with health outcomes.^8,9,10,11,12,13^

There continues to be considerable heterogeneity in the extent, and even existence, of racial disparities in prostate cancer survival outcomes in the literature. Large registry studies have found profound racial disparities in prostate cancer outcomes. Conversely, many studies performed in equal-access settings, such as clinical trials, have shown no difference in outcomes between Black and White men.^14,15,16^ We hypothesized that SDOH are major factors associated with the differences in oncologic outcomes between Black and White men with prostate cancer and that there would be an inverse association between survival outcomes and the extent of disparities in SDOH between races. To better define the association of SDOH and racial disparities in prostate cancer outcomes, we performed the first, to our knowledge, comprehensive meta-analysis of comparative effectiveness research studies in prostate cancer of racial disparities.

## Methods

### Literature Search

In this systematic review and meta-analysis, we performed an electronic search of MEDLINE (via PubMed) on June 5, 2020. Publication dates included in this search ranged from January 1, 1960, to June 5, 2020. The search terms used were *Black* or *African* AND *prostate cancer*. References from identified review articles were added. Studies included in our analysis were required to be conducted among patients within the United States and to perform comparative outcome analysis between Black and White patients. Determination of race for all included studies was based on the self-reported race of the patients included. Restriction to patients within the US was performed to reduce heterogeneity and improve generalizability. End points were required to report time-to-event outcomes (ie, hazard ratios [HRs] based on Kaplan-Meier plots) and not time-specific outcomes (eg, 10-year mortality or odds ratios). The Preferred Reporting Items for Systemic Reviews and Meta-analyses (PRISMA) reporting guideline was followed,^17^ and 2 of us (R.A.V. and D.E.S.) independently reviewed all steps. All conflicts were resolved by consensus of the study team.

### Data Collection

Using predesigned forms, 3 of us (R.A.V., M.B, and J.Q.) independently extracted data. Extracted variables included both study-level and patient-level data. These variables included study design, sample size, patient age, clinical tumor stage, treatment, baseline prostate-specific antigen level, follow-up duration, and National Comprehensive Cancer Network (NCCN) risk grouping. Follow-up duration, the extent of missing data, and the methods used to handle missing data were also collected when available. In addition, statistical methods, including multivariable modeling, propensity scoring, and instrumental variable and sensitivity analysis, were captured. Hazard ratios and 95% CIs were extracted that compared outcomes between Black and White patients.

### End Point Analysis

The primary end point of our study was time to prostate cancer–specific mortality (PCSM), which was typically defined as death attributed to prostate cancer. The secondary end point was overall survival (OS), with death from any cause as the event. Other end points (biochemical recurrence, distant metastasis, and progression-free survival) were prespecified to collect but were not analyzed because of the limited number of studies reporting these end points and the inconsistent definitions.

### Assessment of SDOH

Using definitions from the Healthy People 2030 initiative developed by the US Department of Health and Human Services, we developed an SDOH scoring system.^18^ The scoring system includes variables across all 5 SDOH domains (economic stability; health care access and quality; education access and quality; neighborhood and built environment; and social and community context). All included studies were scored based on predefined criteria to evaluate the association of conditions that influence SDOH with survival outcomes (eTable 1 in Supplement 1). The covariables included in the scoring system were age, comorbidities, insurance status, income status, extent of disease, geography, standardized treatment, and equitable and harmonized insurance benefits. Although age, comorbidities, and extent of disease are not direct measures of SDOH, we included these variables, which are vital to performing comparative outcomes research. In addition, we defined insurance status and equitable and harmonized insurance benefits as separate variables. Insurance status evaluates if studies include whether patients are insured or not; however, equitable and harmonized insurance benefits reflect access to standard-of-care treatment for all patients included in the analysis. The scoring system was discretized into 3 categories: high (≥10 points), intermediate (5-9 points), and low (<5 points) based on the adjustment and accounting of SDOH covariables (eMethods in Supplement 1).

### Statistical Analysis

Meta-analyses for the end points of PCSM and OS were performed with both fixed-effects and random-effects models. The extent of heterogeneity between studies was significant in all meta-analyses, so random-effects models were chosen. Heterogeneity was quantified using both the Cochran *Q* value and the *I*^2^ statistic. Funnel plots were created to assess the risk of publication bias for each end point. Studies were weighted by the inverse variance of the log HRs. Meta-regressions were fit using the restricted maximum likelihood method, and test statistics and 95% CIs were adjusted by the Hartung-Knapp method. Meta-regression models included all studies combined and then separately based on scoring stratification. Meta-regression model coefficients were assessed to evaluate how increasing study-level SDOH score (per 1-unit increase in score) changed the HR across race for PCSM and OS. All statistical analyses were performed using R, version 3.6.2 (R Group for Statistical Computing). All *P* values were from 2-sided tests, and results were deemed statistically significant at *P* < .05.

## Results

### Study Characteristics

**Figure 1.**
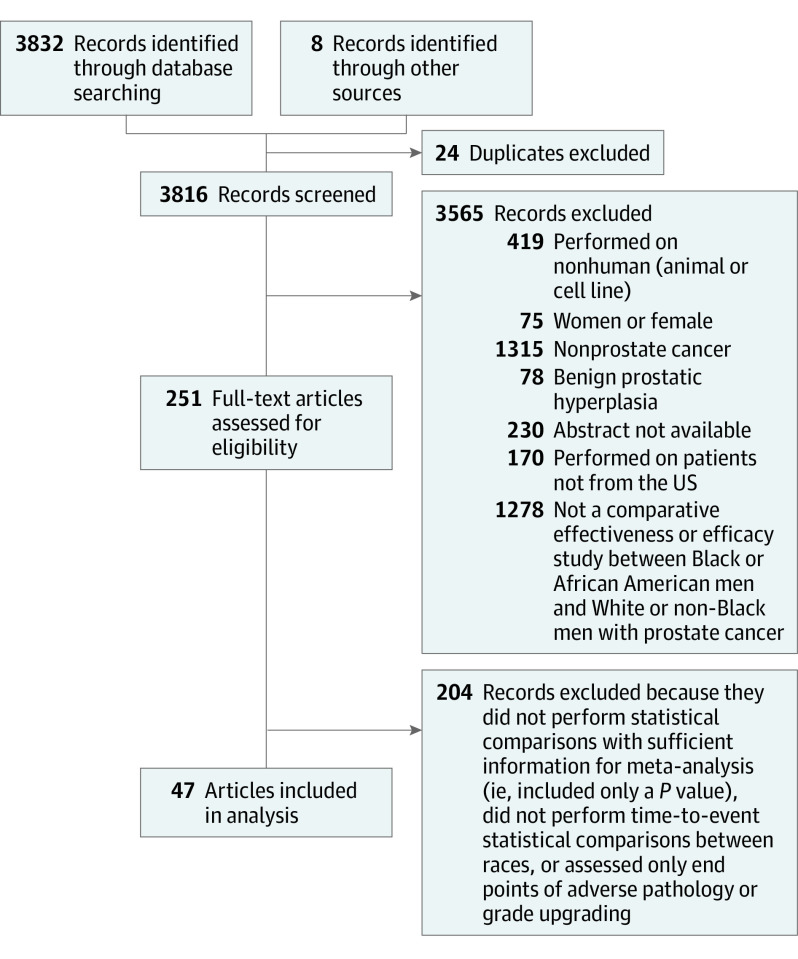
Study Identification and Review of Comparative Effectiveness Research

Our initial search identified 3832 studies (Figure 1). After removing duplicate articles and applying our exclusion criteria, 251 articles were selected for article review. After text review, 47 studies met eligibility criteria and were included in the quantitative analysis; a list of all included studies is in eTable 2 in Supplement 1. Overall, we identified 1 019 908 patients (176 028 Black men and 843 880 White men; median age, 66.4 years [IQR, 64.8-69.0 years]).

Of the 47 studies included, 25 (53.2%) used registry data, 8 (17.0%) were from clinical trials, and the remainder were retrospective single-center or multicenter studies (Table 1).^14,19,20,21,22,23,24,25,26,27,28,29,30,31,32,33,34,35,36,37,38,39,40,41,42,43,44,45,46,47,48,49,50,51,52,53,54,55,56,57,58,59,60,61,62,63,64^ The median follow-up was 66.0 months (IQR, 41.5-91.4 months). The number of published articles conducting comparative analysis of prostate cancer outcomes between races has increased over time, with 19.1% of studies (n = 9) reported between 1960 and 2000, 21.3% of studies (n = 10) reported between 2001 and 2010, and 59.6% of studies (n = 28) reported between 2011 and 2020.

**Table 1.** Study Characteristics

Variable	No. (%) (N = 47)^a^
Study cohort characteristics	
Sample size	
Median (IQR)	3885 (880-16 250)
Mean (SD)	21 082 (49 309)
Patient age, median (IQR), y	66.4 (64.8-69.0)
Follow-up time, median (IQR), mo	66.0 (41.5-91.4)
NCCN risk groups included	
Low	2 (4.3)
Intermediate	2 (4.3)
High	3 (6.4)
Not reported	45 (95.7)
Clinical T stages included	
T1	17 (36.2)
T2	18 (38.3)
T3	14 (29.8)
T4	18 (38.3)
Not reported	22 (46.8)
Study method characteristics	
Data source	
Registry	25 (53.2)
Clinical trial	8 (17.0)
Retrospective	
Single institution	9 (19.1)
Multicenter institution	5 (10.6)
Treatment received reported	
Yes	44 (93.6)
No	3 (6.4)
Reported handling of missing data	
Yes	29 (61.7)
No	19 (40.4)
Statistical adjustments of covariables	
No. of adjustments, median (IQR)	2 (2-4)
Adjusted	
Age	42 (89.4)
Extent of disease	41 (87.2)
Comorbidities	13 (27.7)
Geographic region	8 (17.0)
Insurance status	6 (12.8)
Income	15 (31.9)
Statistical methods	
Multivariable analysis	46 (97.9)
Propensity adjustments	3 (6.4)
Instrumental variable	0
Sensitivity analysis	1 (2.1)
Publication characteristics	
Impact factor	
Median (IQR)	5.7 (4.3-5.9)
Mean	8.7
Publication year	
≤2000	9 (19.1)
2001-2010	10 (21.3)
2011-2020	28 (59.6)

Abbreviation: NCCN, National Comprehensive Cancer Network.

^a^ Percentages within a variable group may add to more than 100% given that an individual study may involve more than 1 category of patients.

### Study Reporting Quality and Statistical Methods

Common baseline characteristics were not routinely reported. Reporting was not performed for NCCN risk groups in 95.7% of studies (n = 45), for clinical T stage in 46.8% of studies (n = 22), and for treatment received in 6.4% of studies (n = 3). Methods for how missing data were handled were not reported by 40.4% of studies (n = 19). Most studies (46 [97.9%]) performed multivariable analysis. However, most studies did not account for known disparities in SDOH between Black and White patients. The median number of covariable adjustments was 2 (IQR 2-4), with adjustments made for comorbidities in 27.7% of studies (n = 13), for insurance status in 12.8% of studies (n = 6), for geographic region in 17.0% of studies (n = 8), and for income in 31.9% of studies (n = 15). Age (42 [89.4%]) and extent of disease (41 [87.2%]) were also not consistently adjusted. Additional statistical methods were rarely used (propensity adjustments in 6.4% of studies [n = 3], instrumental variable in 0%, and sensitivity analysis in 2.1% of studies [n = 1]). Publication bias did not appear to be present for studies included in the PCSM (eFigure 1 in Supplement 1) or OS (eFigure 2 in Supplement 1) analyses.

### Prostate Cancer–Specific Mortality

**Figure 2.**
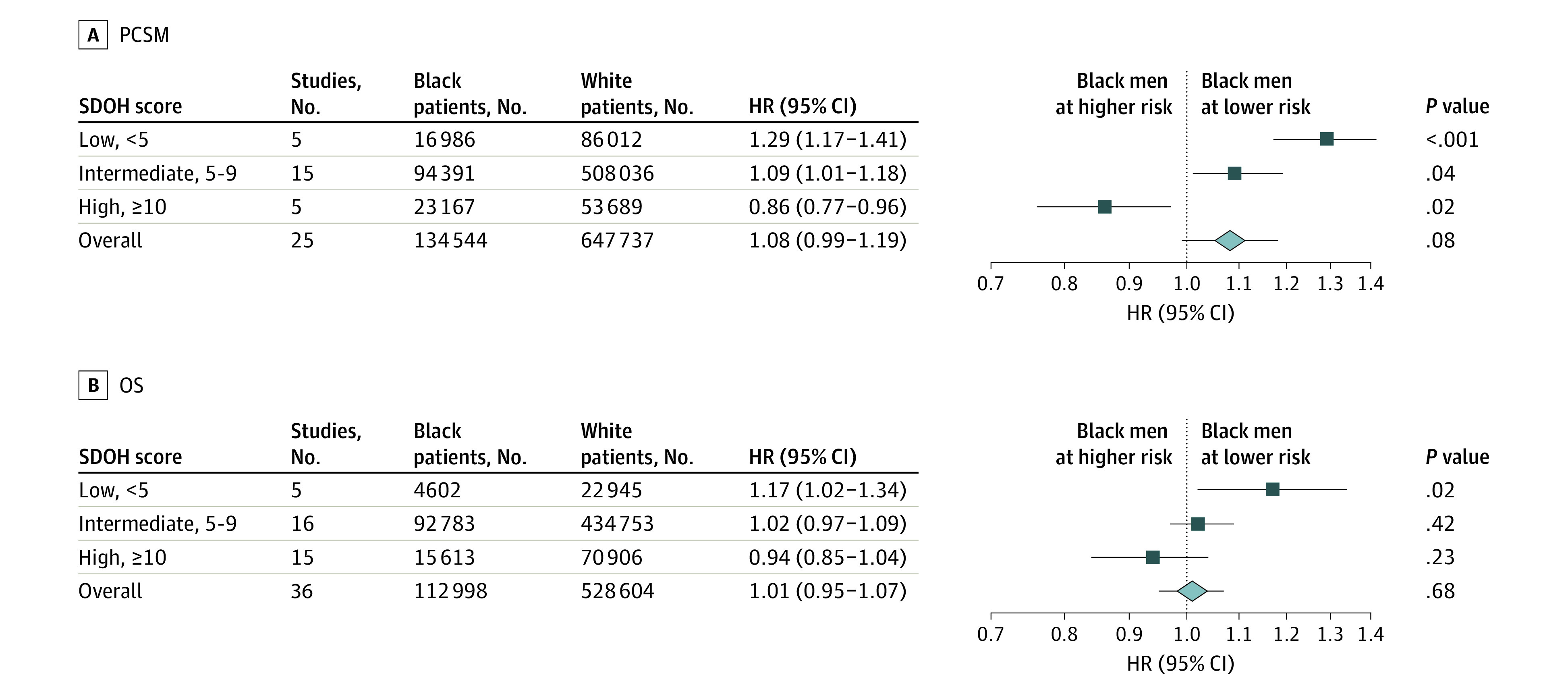
Forest Plot of Prostate Cancer–Specific Mortality (PCSM) and Overall Survival (OS) HR indicates hazard ratio; SDOH, social determinants of health.

Twenty-five studies (53.2%) performed a comparative analysis between Black and White patients with prostate cancer with PCSM as an end point. Pooled estimates found no statistically significant difference in PCSM for Black patients compared with White patients (hazard ratio [HR], 1.08 [95% CI, 0.99-1.19]; *P* = .08) (Figure 2). Five of 25 studies (20.0%), 15 of 25 studies (60.0%), and 5 of 25 studies (20%) had SDOH scores of low, intermediate, and high, respectively. In studies with minimal to no accounting for differences in SDOH variables by race (low SDOH score), Black men had significantly higher PCSM compared with White men (HR, 1.29; 95% CI, 1.17-1.41; *P* < .001). The effect size was smaller for studies with intermediate SDOH scores (HR, 1.09; 95% CI, 1.01-1.18, *P* = .04), and for studies that better accounted for SDOH (high SDOH score), PCSM was significantly lower among Black men compared with White men (HR, 0.86; 95% CI, 0.77-0.96; *P* = .02). Supporting these findings on meta-regression modeling, the regression coefficient was −0.041 (95% CI, –0.059 to 0.023; *P* < .001), demonstrating a significant decrease in PCSM effect size as studies adjusted for more SDOH-related variables (Table 2).

**Table 2.** Meta-Regression of the Association of Race, Social Determinants of Health, and Survival Outcomes

Survival outcome	Estimate (95% CI)^a^	*P* value
Prostate cancer–specific mortality		
Intercept	0.352 (0.212 to 0.492)	<.001
Regression coefficient	−0.041 (−0.059 to 0.023)	<.001
Overall survival		
Intercept	0.158 (0.011 to 0.31)	.04
Regression coefficient	−0.017 (−0.033 to −0.002)	.03

^a^ Negative regression coefficients signify a downward trending association between the incorporation of social determinants of health and hazard ratio of the analyzed end point.

### Overall Survival

Of the 47 studies included, 36 (76.6%) reported OS as a study end point. Meta-analysis of all 36 articles demonstrated that there were no significant differences among Black men compared with White men for OS (HR, 1.01; 95% CI, 0.95-1.07; *P* = .68) (Figure 2). Five of 36 studies (13.9%), 16 of 36 studies (44.4%), and 15 of 36 studies (41.7%) had SDOH scores of low, intermediate, and high, respectively. Studies with less accounting for SDOH (low score) demonstrated a statistically significant difference for Black men compared with White men (HR, 1.17; 95% CI, 1.02-1.34; *P* = .02). There were no significant differences in OS for Black men compared with White men in studies with intermediate SDOH scores (HR, 1.02; 95% CI, 0.97-1.09; *P* = .42) and in studies with high SDOH scores (HR, 0.94; 95% CI, 0.85-1.04; *P* = .23). There was a significant decreasing trend identified with a meta-regression coefficient of −0.017 (95% CI, –0.033 to –0.002; *P* = .03) (Table 2).

## Discussion

The present study, to our knowledge, represents the first comprehensive meta-analysis of studies comparing survival outcomes between Black and White patients with prostate cancer that have been reported in the literature over the past 60 years. Individually, these studies may have supported the historical perception that Black men experience more aggressive and lethal prostate cancer. Collectively, however, after analyzing more than 1 million patients and the interplay of SDOH, race, and survival outcomes, we hope to change the viewpoint from a race-based to a race-conscious framework. We demonstrate a clear association between race, SDOH, and survival outcomes for men with prostate cancer. Studies that poorly accounted for known disparities in SDOH between Black and White men demonstrated significantly higher PCSM for Black patients. In addition, 39.6% of studies included did not report how missing data were handled. Careful consideration of how missing data are handled remains a crucial issue because there has been a clear association between missing registry data and worse cancer outcomes.^65^ In contrast, studies that better accounted for variables that were directly or indirectly associated with SDOH demonstrated that Black men had lower PCSM than White patients. Given the known disparities in SDOH between races in the US, we believe our findings have wide-ranging importance.

Our results align with prior studies that demonstrate that when access to care is equal and treatment is standardized for all patients, Black men have similar or better prostate cancer outcomes.^14^ In a study that incorporated more than 300 000 patients from 3 different cohorts (the Surveillance, Epidemiology, and End Results [SEER] program, Veterans Affairs health system, and National Cancer Institute–sponsored randomized clinical trials), Dess et al^14^ demonstrated that at the population level, Black men had higher PCSM. However, in the Veterans Affairs cohort, which represents the closest model of equal insurance benefits and more equitable access to care in the US, they found no difference in PCSM between races. Furthermore, among patients enrolled in randomized clinical trials in which treatment and follow-up are standardized, Black men had lower PCSM. These results have been recently replicated in another study reported after the date of our literature search.^66^ Ultimately, our results reveal the interplay of societal inequities and prostate cancer outcomes, revealing several potential areas for intervention, starting with how we incorporate variables associated with SDOH into research.

Encouragement of the biomedical community to transition from race-based to race-conscious research is critical. Although hundreds of studies have demonstrated worse health outcomes for Black patients than other races, race is a social construct and not a causal variable or a surrogate for innate biology. Thus, while Black race may be associated with worse health outcomes on a population level, Black patients also are affected by structural racism and disparities in SDOH, regardless of their income class or educational level.^67^ We found an association between differences in SDOH and PCSM for Black men. This association is not causal. However, most of the published literature fails to acknowledge the potential association of racism and SDOH with health outcomes. Vince et al^68^ recently reported that less than 5% of studies throughout history on prostate cancer acknowledged structural racism or disparities in SDOH as potential forms of confounding to their study results, and they often implied that worse outcomes among Black patients were caused by their race. Ultimately, this highlights an overall failure, to this point, among researchers to collect and maintain data sets that include robust information about SDOH and patient outcomes.

Although Black people are more likely to live in high-poverty communities, the association of living in poverty is demonstrated in health outcomes regardless of race. When examining cancer mortality rates in neighborhoods with persistent poverty, Moss et al^69^ found that communities with persistent poverty (≥20% of residents living in poverty for more than 40 years) had significantly higher cancer mortality rates. In addition, when looking at cancer-specific mortality, similar disparities were seen across the various cancers evaluated, which include lung and bronchus, colorectal, breast, prostate, cervical, oropharyngeal, stomach, and liver. These data highlight how poverty, a key component of SDOH, can be associated with the outcomes of patients regardless of race. This is consistent with our findings demonstrating that disparities in survival outcomes for men with prostate cancer are eliminated when even partially accounting for SDOH.

Although the focus of the present study is specifically on prostate cancer, the association of poverty with other health conditions has been documented. Specifically, Tawakol et al^70^ evaluated the association between poverty and cardiovascular disease. In that analysis, patients underwent imaging and assessment of baseline arterial inflammation. No patients at the time of study initiation had a history of cardiovascular disease. The authors found an inverse association between poverty and inflammatory pathway activation, leading to progression of major adverse cardiovascular disease. Thus, there are likely generalizable findings from our work to other cancer types and health conditions that warrant investigation.

### Limitations

This study has some limitations. This was an aggregate meta-analysis of the published literature, and reported studies had heterogenous reporting quality, inclusion criteria, missing data, and follow-up. This heterogeneity, however, reflects the quality of data used throughout history to document survival outcomes between Black and White men with prostate cancer. Individual patient data were not available; thus, duplication of patients from the same data source is possible. Only a small list of variables that influence SDOH were used because no study reported a comprehensive battery of variables associated with SDOH. Thus, the true extent of SDOH and the subsequent association with survival disparities are unknown.

## Conclusions

In this meta-analysis of more than 1 million patients, we found an interaction between race, SDOH, and survival outcomes for men with prostate cancer. When accounting for select established disparities in SDOH, Black men with prostate cancer had similar or improved survival outcomes compared with White men with prostate cancer. These results underscore the importance of accounting for SDOH in racial disparity research. Addressing inequities in SDOH represents modifiable social factors that require attention to reduce the long-standing disparity in prostate cancer health outcomes.

## Notes

Supplement 1. **eFigure 1.** Funnel Plot to Evaluate the Risk of Publication Bias for Prostate Cancer–Specific Mortality End Point **eFigure 2.** Funnel Plot to Evaluate the Risk of Publication Bias for Overall Survival End Point **eTable 1.** Social Determinants of Health Scoring System **eTable 2.** Studies Included in the Meta-analysis
eMethods.

Supplement 2.
Data Sharing Statement
