# Errors in Figure 2

**DOI:** 10.1001/jamanetworkopen.2023.1256

**Published:** 2023-02-13

In the Original Investigation titled “Evaluation of Social Determinants of Health and Prostate Cancer Outcomes Among Black and White Patients: A Systematic Review and Meta-analysis,”^1^ published January 11, 2023, the labels in the forest plot in Figure 2 should be switched in both panels: “Black men at lower risk” should be on the left of the vertical dotted line indicating a hazard ratio of 1.0, and “Black men at higher risk” should be on the right. This article has been corrected.^1^
