# Supplementary material for: Evaluation of Social Determinants of Health and Prostate Cancer Outcomes Among Black and White Patients: A Systematic Review and Meta-analysis
Source: JAMA Netw Open. 2023 Jan 11;6(1):e2250416. doi: 10.1001/jamanetworkopen.2022.50416 (PMC9857531; doi:10.1001/jamanetworkopen.2022.50416)
Supplement: Supplement 1. — eFigure 1. Funnel Plot to Evaluate the Risk of Publication Bias for Prostate Cancer–Specific Mortality End Point eFigure 2. Funnel Plot to Evaluate the Risk of Publication Bias for Overall Survival End Point eTable 1. Social Determinants of Health Scoring System eTable 2. Studies Included in the Meta-analysis eMethods. [file jamanetwopen-e2250416-s001.pdf]

Supplementary Online Content

Vince RA Jr, Jiang R, Bank M, et al. Evaluation of social determinants of health and prostate cancer outcomes among Black and White patients: a systematic review and meta-analysis. *JAMA Netw Open.* 2023;6(1):e2250416. doi:10.1001/jamanetworkopen.2022.50416

eFigure 1. Funnel Plot to Evaluate the Risk of Publication Bias for Prostate Cancer–Specific Mortality End Point
eFigure 2. Funnel Plot to Evaluate the Risk of Publication Bias for Overall Survival End Point
eTable 1. Social Determinants of Health Scoring System
eTable 2. Studies Included in the Meta-analysis
eMethods.

This supplementary material has been provided by the authors to give readers additional information about their work.

© 2023 Vince RA Jr et al. *JAMA Network Open.*

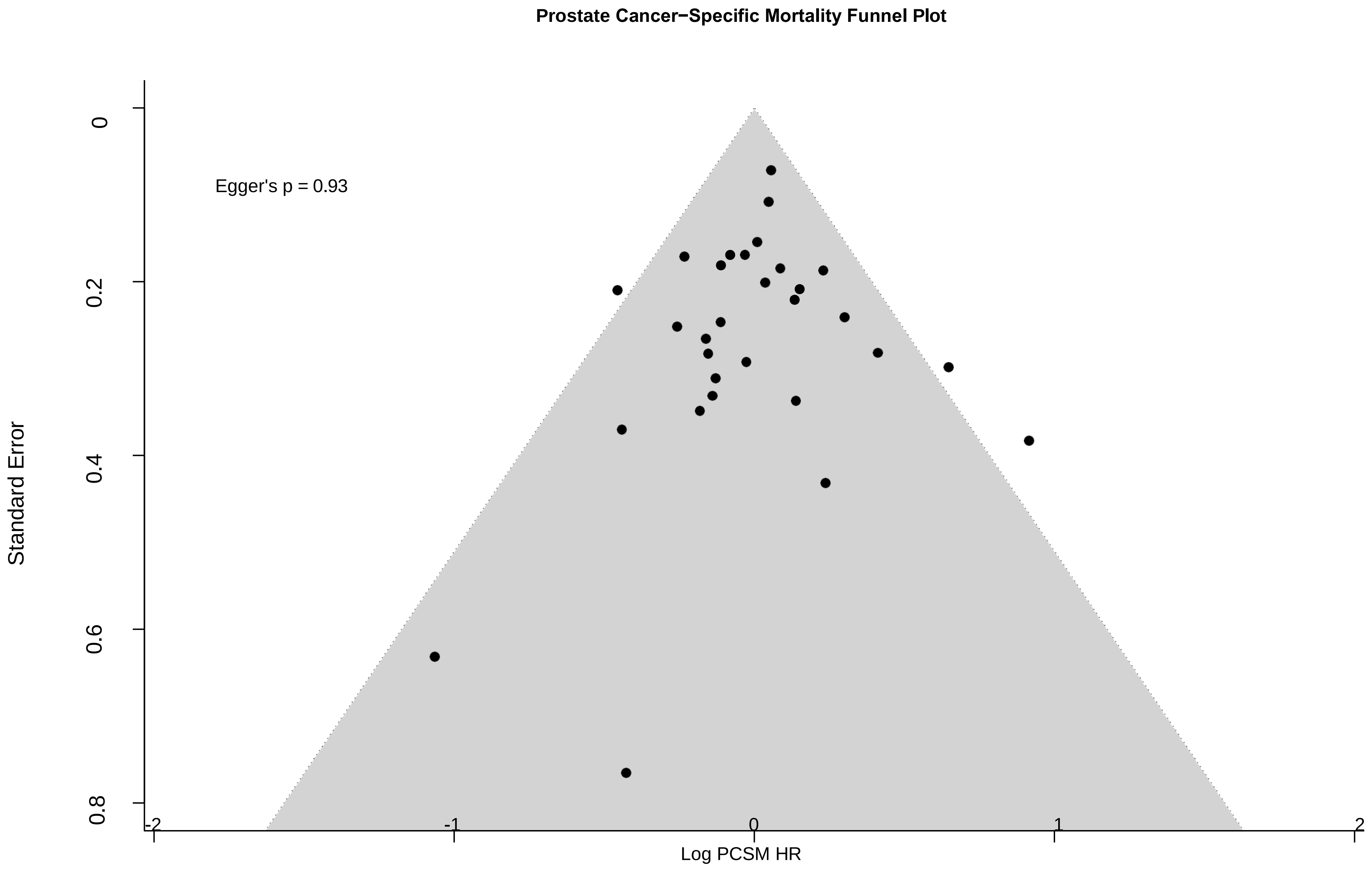

eFigure 1. Funnel Plot to Evaluate the Risk of Publication Bias for Prostate Cancer–Specific Mortality End Point

Abbreviations: PCSM; Prostate Cancer-specific mortality

© 2023 Vince RA Jr et al. *JAMA Network Open.*

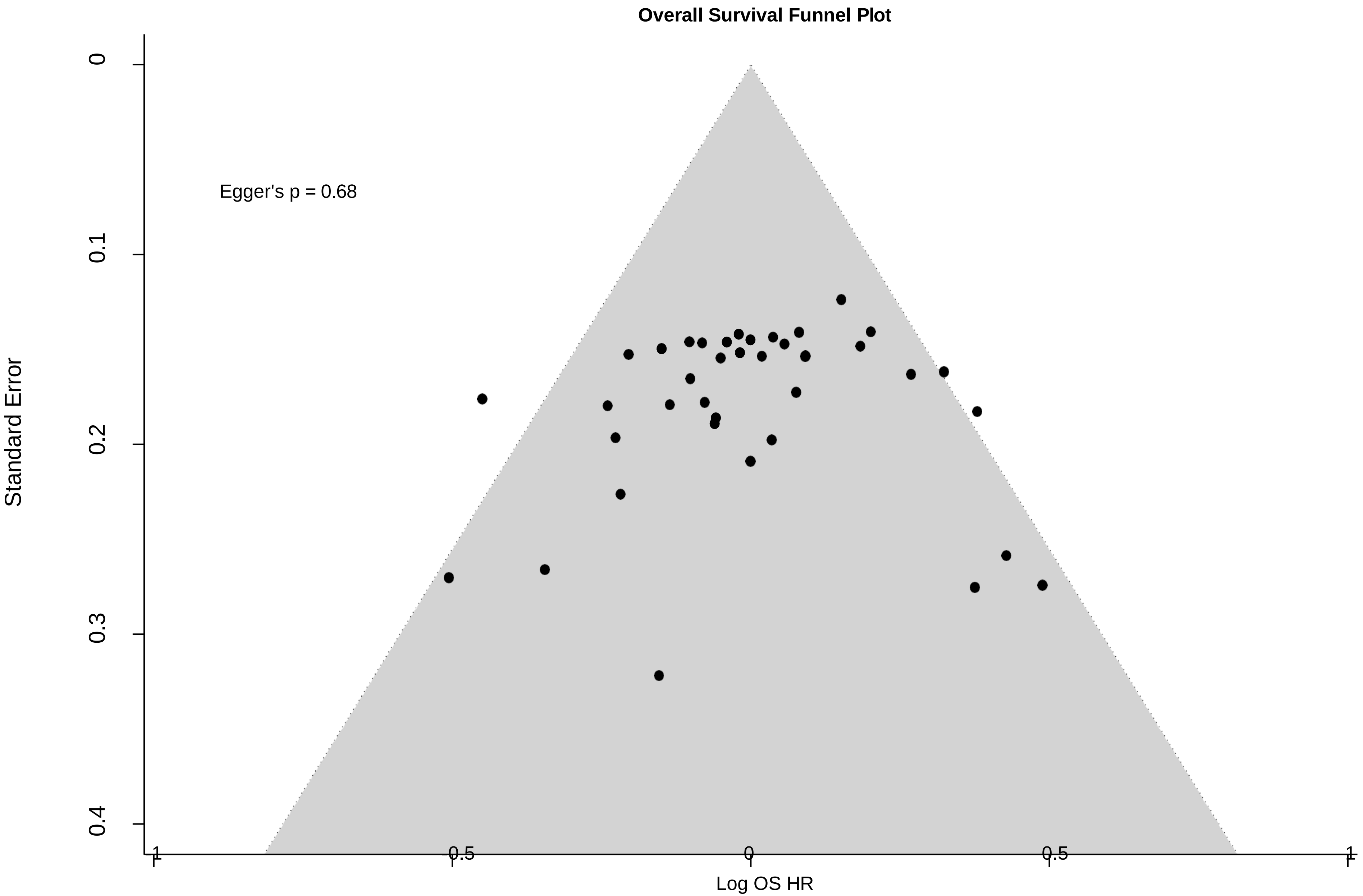

© 2023 Vince RA Jr et al. *JAMA Network Open.*

eFigure 2. Funnel Plot to Evaluate the Risk of Publication Bias for Overall Survival End Point

Abbreviations: OS; overall survival

© 2023 Vince RA Jr et al. *JAMA Network Open.*

eTable 1. Social Determinants of Health Scoring System

The U.S. Department of Health and Human Services started the Healthy People 2030 initiative in 2020. This initiative sets data-driven objectives to improve health and well-being. One of the main pillars of this initiative is social determinants of health. The five domains of social determinants of health as defined by Healthy People 2030 are: economic stability, education access, and quality, healthcare access and quality, neighborhood and built environment, and social and community context.

We selected variables encompassed by the five domains and assigned a scoring system to each variable. The designated scoring system includes the following variables:

1. Disease status: 3 point maximum

   - Diagnostic grade group or Gleason score (1 point)

   - Diagnostic PSA or PSA density (1 point)

   - Clinical or pathologic t-stage (1 point)

2. Age: 1 point
3. Comorbidities: 2 points
4. Insurance status: 2 points
5. Income: 2 points
6. Geography/Region of residency: 1 point
7. Standardized treatment: 5 points
8. Equitable/Harmonized Insurance benefits: 4 points

This 20 point scoring system is designed to evaluate the impact of SDOH on prostate cancer outcomes. We selected to place the highest weight on standardized treatment and equitable/harmonized insurance benefits. These were chosen as these variables are most closely linked to receiving equal treatment despite a patient's race or other social identities. Moreover, data shows inequities in treatment recommendations and receipt of treatment by race. These two environments provide the best opportunity to negate the role of structural racism by providing equitable treatment.

© 2023 Vince RA Jr et al. *JAMA Network Open.*

eTable 2. Studies Included in the Meta-analysis

Study Title	Journal	Year of Publication	Included Endpoints
African-American men and prostate cancer-specific mortality: a competing risk analysis of a large institutional cohort, 1989-2015	Cancer Medicine	2018	PCSM, OS
Association of Black Race with Prostate Cancer- Specific and Other-Cause Mortality	JAMA Oncology	2019	PCSM, OS
Association of census tract-level socioeconomic status with disparities in prostate cancer-specific survival	Cancer Epidemiology, Biomarkers & Prevention	2011	PCSM
Brachytherapy improves outcomes in young men (</=60 years) with prostate cancer: A SEER analysis	Brachytherapy	2017	PCSM
Carcinoma of the prostate: race as a prognostic indicator in definitive radiation therapy	Radiology	1995	PCSM, OS
Determinants of mortality following a diagnosis of prostate cancer in Veterans Affairs and private sector health care systems	American Journal of Public Health	2003	PCSM, OS
Differences in prognostic factors and survival among White men and Black men with prostate cancer, California, 1995-2004.	American Journal of Epidemiology	2007	PCSM
Does race predict the development of metastases in men who receive androgen-deprivation therapy for a biochemical recurrence after radical prostatectomy?	Cancer	2019	PCSM, OS
Factors contributing to the racial differences in prostate cancer mortality	BJU International	2005	PCSM, OS

© 2023 Vince RA Jr et al. *JAMA Network Open.*

Impact of ethnicity on primary treatment choice and mortality in men with prostate cancer: data from CaPSURE	Journal of Clinical Oncology	2010	PCSM
Impact of ethnicity on the outcome of men with metastatic, hormone-sensitive prostate cancer	Cancer	2017	PCSM
Impact of race on survival in patients with clinically nonmetastatic prostate cancer who deferred primary treatment	Cancer	2012	PCSM
Interplay of race, socioeconomic status, and treatment on survival of patients with prostate cancer	Urology	2009	PCSM, OS
Mortality in prostate cancer	Journal of Urology	1996	PCSM, OS
Race and cause specific survival with prostate cancer: influence of clinical stage, Gleason score, age and treatment	Journal of Urology	2000	PCSM
Race and risk of metastases and survival after radical prostatectomy: Results from the SEARCH database	Cancer	2017	PCSM, OS
Race and survival of men treated for prostate cancer on radiation therapy oncology group phase III randomized trials	Journal of Urology	2003	PCSM, OS
Racial disparities in prostate cancer-specific mortality in men with low-risk prostate cancer	Clinical Genitourinary Cancer	2014	PCSM
Racial disparity and socioeconomic status in association with survival in older men with local/regional stage prostate carcinoma: findings from a large community-based cohort	Cancer	2006	PCSM, OS

© 2023 Vince RA Jr et al. *JAMA Network Open.*

Racial/ethnic disparities in survival among men diagnosed with prostate cancer in Texas	Cancer	2011	PCSM
Socioeconomic status, race, and long-term outcomes after radical prostatectomy in an equal access health system: Results from the SEARCH database	Urologic Oncology: Seminars and Original Investigations	2019	PCSM, OS
Surgical control of clinically localized prostate carcinoma is equivalent in African-American and white males	Cancer	1998	PCSM
Survival in blacks and whites after treatment for localized prostate cancer	Cancer	1996	PCSM, OS
Survival of African American and non-Hispanic white men with prostate cancer in an equal-access health care system	Cancer	2020	PCSM
Survival outcomes of radical prostatectomy and external beam radiotherapy in clinically localized high-risk prostate cancer: a population-based, propensity score matched study	Cancer Management and Research	2018	PCSM, OS
The effect of race on survival after local therapy in metastatic prostate cancer patients	Canadian Urological Association Journal	2019	PCSM
Age-race interaction in prostatic adenocarcinoma treated with external beam irradiation.	American Journal of Clinical Oncology	1993	OS
Association between race and oncologic outcome following radical prostatectomy for clinically organ- confined prostate cancer: a long-term follow-up study	World Journal of Urology	2018	OS
Association of African-American ethnic background with survival in men with metastatic prostate cancer	Journal of the National Cancer Institute	2001	OS

© 2023 Vince RA Jr et al. *JAMA Network Open.*

Association of Sociodemographic and Health- Related Factors with Receipt of Nondefinitive Therapy Among Younger Men with High-Risk Prostate Cancer	JAMA Network Open	2020	OS
Clinical Outcomes in Men of Diverse Ethnic Backgrounds with Metastatic Castration Resistant Prostate Cancer	Annals of Oncology	2020	OS
Evaluation of the contribution of demographics, access to health care, treatment, and tumor characteristics to racial differences in survival of advanced prostate cancer	Prostate Cancer and Prostatic Diseases	2018	OS
Impact of race on survival in men with metastatic hormone-refractory prostate cancer	Urology	2004	OS
Improved overall survival trends of men with newly diagnosed M1 prostate cancer: a SWOG phase III trial experience (S8494, S8894 and S9346)	Journal of Urology	2012	OS
Overall Survival of Black and White Men with Metastatic Castration-Resistant Prostate Cancer Treated with Docetaxel	Journal of Clinical Oncology	2019	OS
Race and overall survival in men diagnosed with prostate cancer in the Department of Defense Military Health System, 1990-2010	Cancer Causes and Control	2019	OS
Race and survival following brachytherapy-based treatment for men with localized or locally advanced adenocarcinoma of the prostate	International Journal of Radiation Oncology* Biology* Physics	2011	OS
Race Does Not Affect Survival in Patients with Prostate Cancer Treated with Radiation Therapy	Anticancer Research	2020	OS
Race does not predict the development of metastases in men with nonmetastatic castration- resistant prostate cancer	Cancer	2016	OS

© 2023 Vince RA Jr et al. *JAMA Network Open.*

Race, treatment, and long-term survival from prostate cancer in an equal-access medical care delivery system	JAMA	1995	OS
Racial Discrepancies in Overall Survival among Men Treated with (223) Radium	Journal of Urology	2020	OS
Racial/Ethnic patterns in prostate cancer outcomes in an active surveillance cohort	Prostate Cancer	2011	OS
Stage at presentation and survival of white and black patients with prostate carcinoma	Cancer	1993	OS
Survival after radical prostatectomy	JAMA	1997	OS
Survival of African-American and Caucasian men after sipuleucel-T immunotherapy: outcomes from the PROCEED registry	Prostate Cancer and Prostatic Diseases	2020	OS
The homogeneous and heterogeneous risk factors for the morbidity and prognosis of bone metastasis in patients with prostate cancer	Cancer Management Research	2018	OS
Use of androgen deprivation therapy for metastatic prostate cancer in older men	BJU International	2008	OS
Equivalent racial outcome after conformal radiotherapy for prostate cancer: a single departmental experience	Journal of Clinical Oncology	2001	OS

Studies included in final analysis. Study outcomes listed are the outcomes assess during meta-analysis. Abbreviations; OS: Overall Survival; PCSM: Prostate Cancer Specific Mortality

© 2023 Vince RA Jr et al. *JAMA Network Open.*

eMethods.

We performed an electronic search of MEDLINE (via PubMed) on June 5, 2020. Publication dates included in this search ranged from January 1, 1960, to June 5, 2020. The search terms used were: "black" or "African" AND "prostate cancer". The authors examined all review articles obtained during this search to ensure we included the given references in our analysis. Studies were restricted to those conducted on patients from the United States, include only human subjects, and compare oncologic tumor control outcomes between Black and White patients. During the initial search, a total of 3832 studies were identified. Following the removal of duplicate articles and applying our exclusion criteria, 251 articles were selected for article review. The PRISMA diagram is shown in **Figure 1**. We followed The Preferred Reporting Items for Systemic Review, and Meta-Analyses (PRISMA), and R.A.V and D.E.S. independently reviewed all steps. All conflicts were resolved by consensus.

© 2023 Vince RA Jr et al. *JAMA Network Open.*
