# Supplementary material for: Evaluation of Social Determinants of Health and Prostate Cancer Outcomes Among Black and White Patients: A Systematic Review and Meta-analysis
Source: JAMA Netw Open. 2023 Jan 11;6(1):e2250416. doi: 10.1001/jamanetworkopen.2022.50416 (PMC9857531; doi:10.1001/jamanetworkopen.2022.50416)
Supplement: Supplement 2. — Data Sharing Statement [file jamanetwopen-e2250416-s002.pdf]

Data Sharing Statement

Vince, Jr. Evaluation of Social Determinants of Health and Prostate Cancer Outcomes Among Black and White Patients. *JAMA Netw Open*. Published January 11, 2023.
doi:10.1001/jamanetworkopen.2022.50416

Data
Data available: No
